# Adolescent brain maturation associated with environmental factors: a multivariate analysis

**DOI:** 10.3389/fnimg.2024.1390409

**Published:** 2024-11-19

**Authors:** Bhaskar Ray, Dawn Jensen, Pranav Suresh, Bishal Thapaliya, Ram Sapkota, Britny Farahdel, Zening Fu, Jiayu Chen, Vince D. Calhoun, Jingyu Liu

**Affiliations:** ^1^Tri-Institutional Center for Translational Research in Neuroimaging and Data Science (TReNDS) Georgia State University, Georgia Institute of Technology, Emory University, Atlanta, GA, United States; ^2^Department of Computer Science, Georgia State University, Atlanta, GA, United States; ^3^Neuroscience Institute, Georgia State University, Atlanta, GA, United States

**Keywords:** adolescence, brain development, multivariate, multi-set canonical correlation analysis, structural MRI, functional MRI, environmental factors

## Abstract

Human adolescence marks a crucial phase of extensive brain development, highly susceptible to environmental influences. Employing brain age estimation to assess individual brain aging, we categorized individuals (*N* = 7,435, aged 9–10 years old) from the Adolescent Brain and Cognitive Development (ABCD) cohort into groups exhibiting either accelerated or delayed brain maturation, where the accelerated group also displayed increased cognitive performance compared to their delayed counterparts. A 4-way multi-set canonical correlation analysis integrating three modalities of brain metrics (gray matter density, brain morphological measures, and functional network connectivity) with nine environmental factors unveiled a significant 4-way canonical correlation between linked patterns of neural features, air pollution, area crime, and population density. Correlations among the three brain modalities were notably strong (ranging from 0.65 to 0.77), linking reduced gray matter density in the middle temporal gyrus and precuneus to decreased volumes in the left medial orbitofrontal cortex paired with increased cortical thickness in the right supramarginal and bilateral occipital regions, as well as increased functional connectivity in occipital sub-regions. These specific brain characteristics were significantly more pronounced in the accelerated brain aging group compared to the delayed group. Additionally, these brain regions exhibited significant associations with air pollution, area crime, and population density, where lower air pollution and higher area crime and population density were correlated to brain variations more prominently in the accelerated brain aging group.

## 1 Introduction

During adolescence, the brain experiences rapid development, second only to infancy (Arain et al., 2013). Studies of brain structure using MRI have shown that gray matter exhibits a pre-adolescence increase, followed by a steady decrease into adulthood (Blakemore and Choudhury, 2006). In contrast, white matter density increases roughly linearly until young adulthood (Paus et al., 1999). This general pattern varies across brain regions in terms of rate and time (Giedd et al., 1999; Sowell et al., 2001; Gogtay et al., 2004), and is accompanied by synaptic pruning (Huttenlocher and Dabholkar, 1997), and prolonged increases in myelination (Miller et al., 2012). Functional brain imaging studies also demonstrated that brain responses to stimuli such as sensory inputs, affection, rewards, or demands, change during adolescence (Casey et al., 2005; Laruelle et al., 2002; Kwon et al., 2002; Rubia et al., 2000). Such multidimensional changes in the brain also provide the biological foundation for the maturation of adolescent cognitive ability, which is why cognitive performance is often thought to reflect brain maturation (Dosenbach et al., 2010).

This period of rapid brain maturation has been shown to also be a period of vulnerability to environmental factors (Green et al., 2010). The complex relationship between the brain, behavior, and environmental factors has been well-established (Modabbernia et al., 2021; Xu et al., 2023). Much of the latest research has used the Adolescent Brain and Cognitive Development Study (ABCD) to investigate the effects of environment on brain development. Consistently, researchers using this large adolescent cohort (*N* = 11 k), have found that environmental measures such as neighborhood disadvantage, school environment, and socioeconomic disadvantage are associated with increased functional connectivity (Rakesh et al., 2021, 2023) and reductions in global cortical thickness (Rakesh et al., 2022; Hackman et al., 2021; Taylor et al., 2020). A longitudinal study of the same cohort, looking at these relationships at baseline and year two, found similar associations between changes in brain connectivity and negative environmental factors, suggesting that accelerated maturation of the brain may be an adaptive response to adversity (Brieant et al., 2021).

Beyond the ABCD cohort, other studies have established the relationship between multiple environmental factors, on a variety of scales, with brain maturation, health, and cognitive development. The factors include air pollution (Cipriani et al., 2018), urbanization (Lederbogen et al., 2013; Sampson et al., 2020), negative and unstable family relationships (Bush et al., 2020), and stressful life events (Gapp et al., 2014; Herzberg and Gunnar, 2020). Specifically, on a macro scale, higher air pollution has been associated with a thinner cortex in the precuneus and rostral middle frontal regions, with partially mediating effects on impaired inhibitory control (Guxens et al., 2018), as well as lower functional integration and segregation in key brain networks in school-age children (Pujol et al., 2016). Population density, closely related to urbanicity, has been consistently associated with affective symptoms, including elevated depression (Sampson et al., 2020). Urbanicity is positively correlated with cerebellar volume and negatively correlated with medial prefrontal cortex volume in young people (Xu et al., 2022). Recent research shows that people living in areas with higher ratios of green space exhibit stronger parietal and insular activation during stress, whereas exposure to more air pollution leads to weaker activation in the same brain areas (Dimitrov-Discher et al., 2022). Meso-scale environmental factors like deprived neighborhoods, often characterized by area deprivation, area crime rates, or limited access to school education, are known to have negative effects on young people. These include increased chronic stress (Jorgensen et al., 2023), increased risk of childhood mental health problems, (Baranyi et al., 2021; Sui et al., 2022; Alderton et al., 2019), anxiety, depression (Barnett et al., 2018; Thapaliya et al., 2024), and increased suicidal tendencies (Cairns et al., 2017). Moreover, previous studies on micro-scale environmental factors have established strong links, such as smaller gray matter volume in focal regions resulting from socioeconomic status deprivation (Jeong et al., 2023) and neighborhood disadvantage being associated with lower cortical thickness in brain regions like the cuneus and lateral occipital cortex (Rakesh et al., 2022). A good school environment was also associated with decreased connectivity between the cingulo-opercular network and the default mode network (Rakesh et al., 2023). At the same time, various types of early life adversity were linked to noticeable effects on the brain, including extended activation of prefrontal–hippocampal–amygdala circuits (Smith and Pollak, 2020; Herzberg and Gunnar, 2020).

Despite the depth of our understanding regarding these relationships between environmental factors and specific brain regional alterations, there has been very little research on how they might be related to brain maturation in a holistic way. To estimate the relative brain maturation, neuronal features derived from MRI have been used to create an objective, biological measure, referred to as brain age estimation (Franke and Gaser, 2019; Franke et al., 2010). The gap between estimated brain age and chronological age, referred to as delta age, is commonly used to indicate an individual's brain aging process. Both accelerated and delayed brain aging have been associated with various symptoms of mental illness as well as health conditions (Baecker et al., 2021; Ramduny et al., 2022; Dunlop et al., 2021; Casanova et al., 2022; Phillips et al., 2023). Hereafter, we will refer to the positive/negative gaps between estimated brain age and chronological age as accelerated/delayed brain aging (Peng et al., 2021), indicating the individual differences in brain aging/maturation when compared to the estimated norm of the same age subjects. The validity of brain age estimation has been verified in various cohorts (Baecker et al., 2021) where the brain age gap has been shown to be a reliable biomarker for abnormal brain development, resilient aging process, or risk of mental illness (Franke and Gaser, 2019; Kaufmann et al., 2017; Konrad et al., 2013). In particular, children and adolescent studies, including ours (Ray et al., 2021), have demonstrated that estimated brain age not only reflects age-related changes but is also indicative of cognitive maturation (Ray et al., 2021; Basodi et al., 2021, 2022; Liem et al., 2017; Dosenbach et al., 2010). Accelerated brain age (brain age older than chronological age) is significantly associated with faster information processing speeds and higher verbal comprehension compared to the delayed brain age group (Ray et al., 2023).

Brain age estimation models are effective at selecting brain features that contribute to the accurate estimation of brain age, but they cannot provide coherent information regarding whole brain development patterns. For instance, interrelated bilateral gray matter density in the frontal cortex may both show similar growth patterns, but only one might be selected for the estimation of brain age (Ray et al., 2021). In our previous work, we built a reliable brain age estimation model that identified adolescents in the ABCD Study who were experiencing accelerated or delayed brain maturation (Ray et al., 2023). To fully understand the whole brain patterns related to accelerated vs. delayed brain maturation, as well as investigate the influence of environmental factors, in this study we have applied a multi-set canonical correlation analysis (mCCA) (Li et al., 2009; Zhuang et al., 2020) to overcome the limitations associated with brain age estimation models. Our study is focused on: (i) identifying whole-brain patterns linked to accelerated or delayed brain aging, (ii) exploring the impact of various environmental factors on these brain patterns, (iii) and providing an understanding of how environmental factors may contribute to brain maturation. We seek to reveal, at least partially, the complex interplay between the brain and multi-scale environmental factors, providing further understanding of how brain maturation may be influenced by environments.

## 2 Data and method

### 2.1 Participants

We analyzed the data collected from the ABCD study https://abcdstudy.org (Hagler et al., 2019), which is a 10-year-long study on participants initially recruited at the age of nine to ten from 21 sites across the United States. Along with multisession structural and functional brain MRI scans, the ABCD study also includes key demographic information, including gender, race, socio-economic background, cognitive development, and a mental and physical health assessment of the subjects. Written informed consent from the parents and assent from the child were obtained for each participant, with approval from the Institutional Review Board (IRB). The ABCD dataset is provided by the National Institute of Mental Health Data Archive (NDA) https://nda.nih.gov/. The NDA shares the ABCD data as an open-source dataset, collected from a wide range of research projects across various scientific domains, to enable collaborative science and discovery. In this study, we used data from the ABCD baseline, which contains 11,875 participants, to select a subpopulation with accelerated or delayed brain age.

In our prior study, we developed a refined brain age model to robustly and accurately estimate the brain age of ABCD participants within a narrow age range (9–10 years) (Ray et al., 2024, 2023). The refined model was constructed with two modules. The first module is the brain age model pre-trained with 1,417 subjects aged 8–22 years from the Philadelphia Neurodevelopmental Cohort (PNC) (Satterthwaite et al., 2016), which leveraged the wider age range of PNC data to ensure broader prediction power. After pre-training the first module, the second module was trained by utilizing the ABCD baseline data with a narrow age range (9–10 years) to account for unexplained residual variation (the difference between the prediction of the first module and actual age). The refined brain age estimation was finally obtained by subtracting the estimated residuals from the broader predicted age, thereby improving model accuracy. The final refined model showed the best performance on both ABCD baseline and year-two data with a mean absolute error of 0.49 and 0.48 years, respectively. Furthermore, the brain age gap yielded by the refined model demonstrated significant associations with participants' information processing speed and verbal comprehension ability on the baseline data. In this study, we have applied the model to ABCD full baseline data to estimate participants' brain age. The brain age gaps (estimated brain age—chronological age) were computed (see Supplementary Figure 1) and were found to roughly follow a normal distribution. We identified 7,435 participants whose brain age gap was 0.41 standard deviation away from the mean after z transformation, partitioned into accelerated brain age group (*z*>0.41:*N* = 3, 755) and delayed brain age group (*z* < −0.41:*N* = 3, 680). The threshold of 0.41 was chosen to include roughly the bottom and top third of the cohort. In our analysis, we specifically targeted these two subgroups to identify brain patterns with significant differences between the two groups and linked to environmental factors. We did not use all participants to emphasize the factors contributing most to the variations between accelerated and delayed brain age. In the data quality control steps, we removed samples following the inclusion recommendation of ABCD release version 5 for T1 weighted sMRI and rs-fMRI and removed samples with missing values in the environmental factors. Finally, we have 4,264 samples (Accelerated group: *N* = 2,149, Delayed group: *N* = 2,115) for our analysis. The two groups listed in Table 1 had no significant differences in age, gender, and race.

**Table 1:** Demography table.

**Demographics**	**Accelerated (*N* = 2,149)**	**Delayed (*N* = 2,115)**
Mean age	9.94 ± 0.63	9.93 ± 0.63
Gender	1,158 male/991 female	1,012 male/1,103 female
Race	1,714 white/412 black	1,678 white/334 black
		92 other/34 missing	
Mean total composite score	87.94 ± 8.71	86.94 ± 8.65
Mean fluid composite score	93.32 ± 0.29	92.13 ± 10.08
Mean crystallized composite score	87.48 ± 6.79	87.02 ± 6.84

### 2.2 sMRI data preprocessing and feature generation

We extracted three types of brain features: (i) gray matter density of 100 independent components derived from independent component analysis (ICA) (Xu et al., 2009) of gray matter images, (ii) 152 brain morphological features derived from FreeSurfer version v5.3 (Khan et al., 2008), and (iii) 1,378 static functional network connectivity (sFNC) values (Saha et al., 2022) derived from resting state functional MRI.

Gray matter (GM) density maps were generated by preprocessing the T1-weighted sMRI images using the Statistical Parametric Mapping 12 (SPM12) (Ashburner et al., 2014) software toolbox. Six types of tissue maps (gray matter, white matter, CSF, bone, soft tissue, and others) were created in Montreal Neuroimaging Institute (MNI) space after jointly segmenting and spatially normalizing the T1-weighted sMRI images using SPM12 default tissue probability maps. The gray matter maps were then smoothed using a 6 *mm*^3^ Gaussian kernel. Finally, we applied quality control on the individual gray matter maps and selected those correlated to the group mean gray matter map at correlation ≥0.9. After quality control, the gray matter maps were then masked to only include voxels with a gray matter density value >0.2. We then applied independent component analysis (ICA) (Bell and Sejnowski, 1995; Amari, 1998) to extract 100 brain components. ICA decomposes the gray matter data into a linear combination of maximally independent components, called source-based morphometry (SBM) (Xu et al., 2009). Each component as a brain network identifies a network of voxels with covarying gray matter patterns and often these components resemble those in resting fMRI data (Luo et al., 2020). The ICA loadings reflect how these brain networks are expressed across subjects, which are used as one type of brain feature in this study, referred to as gray matter density of 100 independent components. In addition, the brain morphological measures derived by FreeSurfer version v5.3 (Khan et al., 2008) are provided by the ABCD study (data release version 5). We selected 152 measures as the second type of brain features, which included the estimated total intracranial volume, cortical thickness and cortical volume, and subcortical volume of the human brain based on the Desikan atlas (Desikan et al., 2006).

### 2.3 fMRI data preprocessing and functional network connectivity (sFNC) features

We conducted preprocessing on the raw resting-state fMRI data using a combination of the FMRIB Software Library (FSL) v6.0 (Jenkinson et al., 2012; Smith et al., 2004; Woolrich et al., 2009) toolbox and the Statistical Parametric Mapping (SPM) 12 toolbox within the MATLAB 2020b environment. The preprocessing steps involved: (i) correcting for rigid body motion; (ii) addressing distortion; (iii) eliminating dummy scans; (iv) normalizing the data to standard Montreal Neurological Institute space; and (v) applying smoothing with a 6 mm Gaussian kernel. We conducted data quality control on the preprocessed fMRI data using the Neuromark framework (Fu et al., 2023). The FSL MCFLIRT tool was employed to rectify any rigid body motion observed in the subject's head during the fMRI scanning. After correcting head motion, distortion correction in the fMRI images was performed using field map files. These files were obtained by acquiring phase encoding in both the anterior-posterior (AP) and posterior-anterior (PA) directions with the FSL tool topup. The distortion present in the fMRI volume was then addressed by applying the output field map coefficients using the FSL tool applytopup. After distortion correction, 10 initial scans with significant signal changes were discarded to help the tissue gain a steady state of radiofrequency excitation. Then wrapping and smoothening of the fMRI data were done with MNI space 3 × 3 × 3 *mm*^3^ spatial resolution and a Gaussian kernel with a full width at half maximum (FWHM) of 6 mm. We employed the *Neuromark*_*fMRI*_1.0 (Du et al., 2020) network templates to extract intrinsic connectivity networks (ICNs) and corresponding time courses (TCs) through an entirely automated spatially restricted ICA method. These templates were obtained based on replicated networks estimated from two healthy control datasets, the human connectome project (Van Essen et al., 2012) and the genomics super struct project (Holmes et al., 2015). More details of the Neuromark framework and templates can be found in the GIFT toolbox http://trendscenter.org/software/gift and at http://trendscenter.org/data (Correa et al., 2005). These spatial priors have been established to be highly consistent between pipelines and across various datasets and populations. We obtained 53 intrinsic connectivity networks for each subject by implementing this approach. To address any confounding effects, such as the greater degree of head motion present in pediatric images, we included four additional post-processing steps to regulate the remaining noise in the TCs of ICNs. These steps involved detrending linear, quadratic, and cubic trends, eliminating detected outliers, implementing multiple regression on the head motion parameters, and bandpass filtering. After the post-processing phase, Pearson correlation coefficients between post-processed TCs were calculated to measure the static functional network connectivity (sFNC) among 53 ICNs. The 53 × 53 symmetric sFNC matrix was then flattened, and 1,378 correlation values from off-diagonal elements were extracted. These form the third type of brain features of this study.

### 2.4 Environmental factors

In our study, we utilized a total of nine environmental factors spanning macro, meso, and micro scales (Thapaliya et al., 2021, 2023) from the ABCD Cohort, both because of their established relevance in the literature and our own group's previous work (Thapaliya et al., 2021, 2024) with these factors elucidating the complex relationship between the brain and the environment. We are aware that more environmental variables are now available, which are unexplored in our current research and can be utilized in future studies. The factors we included are air pollution, population density, area crime, neighborhood safety, school safety, household income, family conflict, early life stress (ELS), and area deprivation. Each factor was derived by aggregating multiple related variables to construct a comprehensive measure through summation, with variables reversed, if necessary, to maintain consistency in the direction of the effect. A higher value in air pollution, population density, area crime, family conflict, ELS, and area deprivation implies a negative or unfavorable direction. Conversely, higher values in neighborhood safety, school safety, and household income indicate a positive or desirable direction, such as a safe neighborhood and school environment and better socio-economic status. A detailed explanation of the relevant variables of each environmental factor is presented in Supplementary material.

### 2.5 Cognitive measures

In this study, we utilized three cognitive composite scores from the NIH Toolbox Cognition Battery (Akshoomoff et al., 2013) for our analysis. The three main cognitive summary scores are: (i) crystallized cognition composite score (combination of picture vocabulary and oral reading recognition tests), which reflects crystallized cognition based on past learning experiences. (ii) fluid cognition composite score (includes tests that evaluate fluid abilities like executive function, working memory, attention, and processing speed), which demonstrates the ability for new learning and information processing in unexplored situations. (iii) total cognitive function composite score, which provides a comprehensive measure of general cognitive abilities by combining both the crystallized and fluid cognition composite scores.

### 2.6 Statistical analysis

We utilized multi-set canonical correlation analysis to identify the distinct multivariate patterns of different brain feature sets, as well as environmental factors, for the accelerated and delayed subpopulation. Canonical correlation analysis (CCA) is a statistical approach, first proposed by Hotelling (Hardoon et al., 2004) in 1936, which tries to find pairs of linear projections for different views in such a way that the correlation between them is maximized.

If we have two data matrices $X=\left[{\text{x}}_{1},{\text{x}}_{2},\ldots ,{\text{x}}_{n}\right]\in {\mathbb{R}}^{d_{x}\times n}$ and $Y=\left[{\text{y}}_{1},{\text{y}}_{2},\ldots ,{\text{y}}_{n}\right]\in {\mathbb{R}}^{d_{y}\times n}$ where *n* denotes the number of samples and *d*_*x*_, *d*_*y*_ indicate feature dimensions for data matrices *X* and *Y*, respectively. CCA will find *m* pairs of linear projections. $W_{x}=\left[{\text{w}}_{\text{x},1},{\text{w}}_{\text{x},2},\ldots ,{\text{w}}_{x,m}\right]\in {\mathbb{R}}^{d_{x}\times m}$ and $W_{y}=\left[{\text{w}}_{\text{y},1},{\text{w}}_{\text{y},2},\ldots ,{\text{w}}_{y,m}\right]\in {\mathbb{R}}^{d_{y}\times m}$ represent the canonical weight matrices for two data matrices *X* and *Y*. The correlation between *a*^th^ pair of canonical projections ${\text{w}}_{x,a}^{T}X$ and ${\text{w}}_{y,a}^{T}Y$ are maximized as in Equation 1. Equation 1 can be simplified as Equation 2.


(1) $$\begin{array}{l} \rho \left(X^{T}w_{x,a},Y^{T}w_{y,a}\right)=\frac{w_{x,a}^{T}XY^{T}w_{y,a}}{\sqrt{\left(w_{x,a}^{T}XX^{T}w_{x,a}\right)\left(w_{y,a}^{T}YY^{T}w_{y,a}\right)}} \end{array}$$

(2) $$\begin{array}{l} \begin{array}{c} \underset{w_{x,a}w_{y,a}}{\max}w_{x,a}^{T}XY^{T}w_{y,a}\\ \text{s}.\text{t}.w_{x,a}^{T}XX^{T}w_{x,a}=1,\;w_{y,a}^{T}YY^{T}w_{y,a}=1,\\ \left(\text{orthogonality constraint}\right)\\ w_{x,a}^{T}XX^{T}w_{x,b}=0,\;w_{y,a}^{T}YY^{T}w_{y,b}=0\;\forall a\neq b:a,b\in \left\{1,2,\ldots ,m\right\} \end{array} \end{array}$$

The mCCA approach basically extends the concept of the general form of CCA in order to find correlated patterns among more than two views. Multi-view CCA aims to maximize the sum of pairwise canonical correlations via optimizing canonical vectors of all views. *c*_*i*_ is the regularization parameter to be defined.


(3) $$\begin{array}{l} \begin{array}{c} w_{opt}=\underset{w}{\mathrm{argmax}}\left\{\underset{i}{\sum }\underset{j\neq i}{\sum }w_{i,a}^{T}X_{i}^{T}X_{j}w_{j,a}\right\}\\ \text{s}.\text{t}.\left(1-c_{i}\right)w_{i,a}^{T}X_{i}^{T}X_{i}w_{i,a}+c_{i}w_{i,a}^{T}w_{i,a}=1,\\ \left(\text{orthogonality constraint}\right)\\ w_{i,a}^{T}X_{i}^{T}X_{i}w_{i,b}=0\;\forall i,\forall a\neq b:a,b\in \left\{1,2,\ldots ,m\right\} \end{array} \end{array}$$

In Equations 2, 3, the orthogonality constraint ensures that every pair of canonical variables is orthogonal/uncorrelated with another pair of canonical variables.

To investigate the covariation patterns of the brain and the environment in this subpopulation, we conducted a 4-way mCCA analysis using the environmental factors and three types of brain features. Specifically, mCCA was applied to the subject gray matter loadings of 100 independent components, 152 morphological measures, 1,378 sFNC values, and nine environmental factors. The data were split as follows: 80% for training and 20% for testing, with a stratified 3-fold cross-validation in the training set to select the *c*_*i*_ parameter and avoid overfitting issues. We used the CCA-Zoo (Chapman and Wang, 2021) package for our analysis. To be conservative, we selected the number of canonical variable sets based on the smallest dataset. Since the top four principal components explained nearly 90% of the variance in the smallest input data matrix of nine environmental factors, we applied mCCA with four sets of canonical variables in the training data to derive correlated brain and environmental variables. Thus, only those brain patterns potentially associated with environmental factors were extracted. The derived latent variables were directly projected into the testing data to verify their associations. In order to test for significant mean differences between the accelerated and delayed groups, we applied the analysis of variance (ANOVA) method on latent variables. To help further understanding of our findings, we examined the canonical weight and shared variance percentage of each original feature to identify its contribution to the canonical latent variables. Canonical weights are the values in the canonical vectors W in Equation 3. The shared variance percentage is calculated as the square of the correlation between an original observed feature and its corresponding canonical variable (r2).

To test the stability and robustness of the mCCA results, we applied the mCCA analysis 100 times, each time randomly splitting the data into an 80/20 split for training and hold-out test data. The mCCA model was trained on the training data and tested on the hold-out data. We then reported the average canonical weights, the variance explained, and the frequency of a given feature that was among the “Top 5” list across the 100 mCCA models on the hold-out test data. For illustration purposes, related brain areas for the top five (arbitrary selection) features based on the shared variance percentage from the three brain datasets (ICA, FreeSurfer, and sFNC) are plotted using Talairach Daemon software from the GIFT toolbox and the Desikan atlas and displayed in the results section (Rachakonda et al., 2007).

To test the associations with cognition and brain maturation group, we implemented linear mixed-effects regression models using cognitive measures (NIH Toolbox Fluid Cognition Composite Score, Crystallized Cognition Composite Score, and Total Cognition Composite Score) as the dependent variable, with age, gender (Male = 0, Female = 1), and brain maturation group (Accelerated = 3, Delayed = 2) as fixed-effect independent variables. ABCD Family ID nested within ABCD site information was included as random-effect variables. We included each subject's actual age and gender as predictors, along with the developmental group, to control for the known effects of age and gender on cognition during development. Additionally, we conducted linear mixed-effects regression analyses on our 20% test data to examine associations with cognitive measures and brain-related canonical variables, using the cognitive score as the dependent variable and age, gender, and brain-related canonical variables as fixed-effect independent variables, while ABCD Family ID nested within ABCD site information were included as random effect variables.

## 3 Results

In our 4-way mCCA analysis, four sets of canonical variables were extracted during the training, while each set comprised of four canonical latent variables: three latent brain variables and one latent environmental variable. All four canonical variables within each set were significantly correlated (*p*-values that survived FDR correction) during the training phase. The direct projection of derived latent variables into the 20% hold-out testing data revealed that all four sets of canonical latent variables were significantly cross-correlated within each set in the testing data as well (see Table 2). Since results from the testing data are more impartial, hereafter we present only those results. The correlations between canonical variables of the brain features were high with r-values ranging from 0.88 to 0.55. In contrast, the correlations of environmental factors with brain features were low, but statistically significant, with *r*-values ranging between 0.37 and 0.09.

**Table 2:** Pairwise correlation of four sets of canonical variables (results from the 20% testing data).

**Pairwise correlation**	**Canonical variables**			
		**SET 1 (*r*, *p*)**	**SET 2 (*r*, *p*)**	**SET 3 *r*, *p***	**SET 4 *r*, *p***
ICA GM-FreeSurfer	*r* = 0.88, *p* < 1e-16	r = 0.77, *p* < 1e-16	r = 0.84, *p* < 1e-16	r = 0.80, *p* < 1e-16
ICA GM-sFNC	*r* = 0.64, *p* < 1e-16	*r* = 0.65, *p* < 1e-16	*r* = 0.64, *p* < 1e-16	*r* = 0.63, *p* < 1e-16
ICA GM-environment	*r* = 0.37, *p* < 1e-16	*r* = 0.15, *p* < 1e-16	*r* = 0.17, *p* < 1e-16	*r* = 0.11, *p* = 9e-4
FreeSurfer-sFNC	*r* = 0.64, *p* < 1e-16	*r* = 0.68, *p* < 1e-16	*r* = 0.55, *p* < 1e-16	*r* = 0.60, *p* < 1e-16
FreeSurfer-environment	*r* = 0.36, *p* < 1e-16	*r* = 0.18, *p* < 1e-16	*r* = 0.14, *p* = 1e-4	*r* = 0.09, *p* = 1.2e-2
sFNC-environment	*r* = 0.34, *p* < 1e-16	*r* = 0.16, *p* < 1e-16	*r* = 0.20, *p* < 1e-16	*r* = 0.12, *p* = 4e-4

A detailed report of the brain regions and associated environmental variables is presented in Supplementary Tables 1–8. The 1st derived environmental variable highlights the positive effect of household income (shared variance percentage of 71.95%, canonical weight of 0.16) and the negative effect of area deprivation (shared variance percentage of 68.56%, canonical weight of −0.14) (see Supplementary Table 2). The 2nd environmental variable highlights air pollution and area crime (see Supplementary Table 4). The 3rd environmental variable emphasizes area deprivation (shared variance percentage of 77.11%, canonical weight of 0.12) in contrast to household income (shared variance percentage of 44.15%, canonical weight of −0.02) and neighborhood safety (shared variance percentage of 44.95%, canonical weight of −0.04) (see Supplementary Table 6). The 4th environmental variable highlights relatively small effects of negative household income (shared variance percentage of 64.16%, canonical weight of −0.10) and positive area deprivation (shared variance percentage of 49.04%, canonical weight of 0.06) (see Supplementary Table 8). All four environmental variables are associated with specific brain features (see Supplementary Tables 1–8).

**Figure 1:**
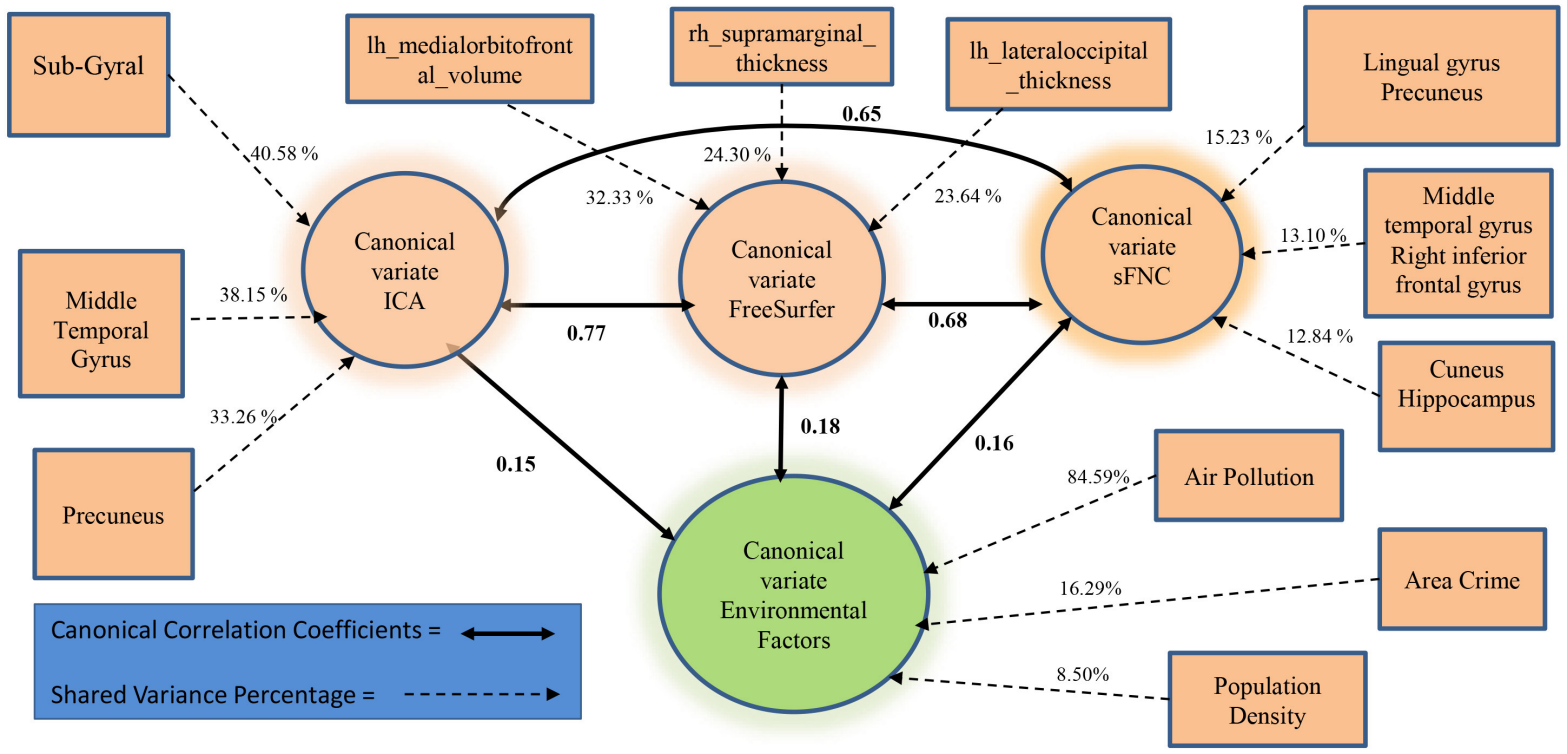
The association among the 2nd set of canonical variables from 20% testing data.

**Figure 2:**
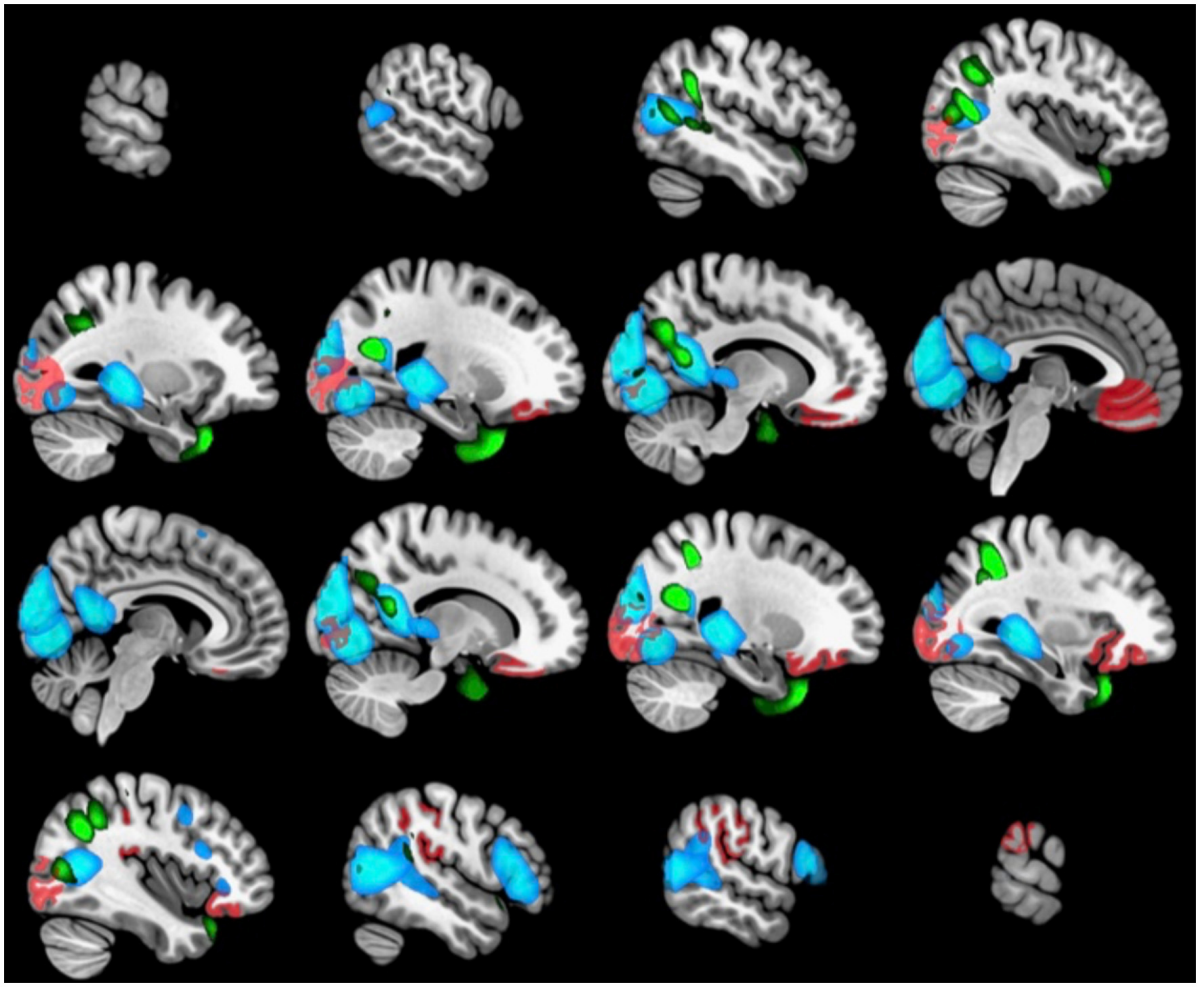
The brain regions associated with the top five contributing features of the 2nd set of brain canonical variables (ICA GM in green, FreeSurfer in red, sFNC in blue).

We further examined whether the derived canonical variables had differences between the accelerated and delayed brain age groups. The 2nd set of canonical variables showed consistently significant differences in the three brain-related canonical latent variables, where the accelerated group had higher values than the delayed group. The environmental canonical latent variable did not show group differences in all four sets. We presented the results of the testing data in Table 3. Given our interest is in identifying brain patterns related to brain development, the 2nd set of canonical variables became our focus hereafter. We presented their four-way associations on testing data in Figure 1. The figure displays the pairwise canonical correlation coefficients among the four canonical latent variables, as well as the top three features contributing to each of the canonical variables for illustration purposes. Based on the percentage of shared variance calculated using the testing data, we have identified the top five contributing features for each canonical variable. Table 4 provides feature names, canonical weights, and shared variance percentages. The resulting brain areas corresponding to these top five features are depicted collectively in Figure 2, with green indicating gray matter ICA components, red representing FreeSurfer morphological features, and blue denoting sFNC.

**Table 3:** Group difference of four sets of canonical variables on the 20% testing data.

**Canonical variables**	**Group differences of canonical variables**			
	**SET 1 (*F*, *p*)**	**SET 2 (*F*, *p*)**	**SET 3 (*F*, *p*)**	**SET 4 (*F*, *p*)**
ICA GM	*F* = 12.67, *p* = 3.92e-4	*F* = 17.57, *p* = 3.1e-5	Not significant	Not significant
FreeSurfer	Not significant	*F* = 22.93, *p* = 2e-6	*F* = 10.70, *p* = 11.11e-3	Not significant
sFNC	Not significant	*F* = 18.67, *p* = 1.7e-5	Not significant	Not significant
Environment	Not significant	Not significant	Not signifi cant	Not significant

**Table 4:** The top five contributing features of the 2nd set of canonical variables, along with their canonical weights and shared variance percentages in the testing data, are presented.

**Canonical variates**	**Top 5 contributing features**	**Canonical weights**	**Shared variance percentages (r2%)**	**Stability test results (mCCA 100 iterations)**		
				**Canonical weights mean (95% CI)**	**Shared variance percentages mean (95% CI)**	**Frequency within the top 5 list**
ICA GM	Sub-gyral 1	0.15	40.58	0.15 (0.126, 0.165)	40.47 (39.95, 40.99)	100%
	Middle temporal gyrus 1	−0.06	38.15	−0.05 (−0.055, −0.038)	38.25 (37.69, 38.80)	100%
	Precuneus	−0.01	33.26	−0.01 (−0.007, −0.005)	33.12 (32.54, 33.71)	100%
	Middle temporal gyrus 2	−0.07	27.36	−0.06 (−0.068, −0.049)	26.54 (25.98, 27.11)	85%
	Superior temporal gyrus	0.03	26.69	0.03 (0.024, 0.029)	25.63 (25.04, 26.23)	72%
FreeSurfer	Left medial Orbitofrontal Volume	−0.10	32.33	−0.08 (−0.096, −0.067)	32.76 (32.21, 33.31)	100%
	Right supramarginal thickness	0.05	24.30	0.06 (0.051, 0.075)	23.89 (23.33, 24.46)	98%
	Left lateral occipital thickness	0.09	23.64	0.08 (0.066, 0.088)	23.70 (23.23, 24.16)	100%
	Right lateral occipital thickness	0.07	22.73	0.06 (0.047, 0.067)	21.42 (20.87, 21.97)	86%
	Right lateral orbitofrontal volume	−0.08	20.78	−0.07 (−0.076, −0.059)	20.56 (20.03, 21.10)	96%
sFNC	Lingual gyrus, precuneus	0.02	15.23	0.02 (0.015, 0.021)	14.91 (14.53, 15.29)	100%
	Middle temporal gyrus, right Inferior frontal gyrus	−0.02	13.10	−0.02 (−0.019, −0.013)	12.86 (12.51, 13.21)	98%
	Cuneus, hippocampus	0.02	12.84	0.02 (0.012, 0.019)	13.02 (12.64, 13.41)	93%
	Cuneus, precuneus	0.003	12.25	0.005 (0.004, 0.006)	11.95 (11.62,12.27)	87%
	Lingual gyrus, hippocampus	0.009	12.25	0.01 (0.007, 0.009)	12.39 (12.02, 12.76)	82%
Environmental factors	Air pollution	−0.26	84.59	−0.25 (−0.280, −0.216)	85.04 (84.41, 85.66)	100%
	Area crime	0.06	16.29	0.05 (0.039, 0.055)	16.59 (15.71, 17.47)	100%
	Population density	0.01	8.50	0.01 (0.004, 0.008)	8.45 (7.94, 8.96)	100%
	Family conflicts	0.04	1.92	0.03 (0.025, 0.036)	2.23 (1.90, 2.57)	89%
	Neighborhood safety	0.01	1.01	0.01 (0.005, 0.009)	1.19 (0.90, 1.48)	77%

The stability test results show the mean and 95% confidence intervals for the canonical weights and shared variance percentages of the top features, as well as their frequency within the top five list of features across 100 iterations of mCCA on hold-out test data.

We performed linear mixed-effect regression model analyses on the accelerated and delayed brain age group to test the associations with cognitive measures and the brain maturation group. The results showed a *t*−*value*:4.24, *p* < 1*e*−16 for the Total composite score, *t*−*value*:4.19, *p* < 1*e*−16 for the Fluid composite score, and *t* = 2.63, *p* = 0.009 for the Crystallized composite score, suggesting the accelerated age group showed significantly higher performance on cognitive tests, consistent with accelerated brain maturation. Moreover, the association analyses between cognitive scores and brain-related canonical variables showed that the 1st set of brain canonical variables were all significantly associated with all three cognitive composite scores, passing FDR correction. Sets 2, 3, and 4 brain canonical variables were not associated with cognitive measures. The detailed association results are listed in Supplementary Table 9.

## 4 Discussion and conclusion

Our study aims to explore the multifaceted brain features that are associated with brain maturation and their relationship with environmental factors. From the ABCD cohort, we have identified a subpopulation of participants with either accelerated or delayed brain age using the brain age estimation model from our prior study. These two groups showed significant differences in cognitive measures, with the accelerated brain age group performing higher than the delayed group on cognitive measures, even after adjusting for biological age. To study environmental effects on multimodal brain features, we performed a 4-way mCCA analysis on regional gray matter density, morphometric measures, resting-state functional network connectivity, and environmental factors from nine domains. The 4-way mCCA analysis revealed highly related patterns between the three types of brain measures and their relationship with the environment.

Four sets of 4-way canonical variable correlations were verified (all four latent variables were significantly correlated in the testing data), and each set highlights a different environmental association with brain patterns. While the 1st, 3rd, and 4th sets present mainly effects from household income, area deprivation, and neighborhood, the 2nd set presents effects of air pollution and area crime at large. The 2nd set of brain canonical variables also demonstrates significant mean differences between the accelerated and delayed groups. The 1st set of brain canonical variables are also positively associated with all three cognitive scores (see Supplementary Table 9). Given they relate significantly to household income, which is one of the most studied environmental factors with abundant evidence for associations with brain and cognition (Tomasi and Volkow, 2021, 2023), we are not surprised to observe this particular set of brain canonical variables associated with overall cognition. The lack of cognitive association of other sets of brain variables warrants further investigation on more specific cognitive ability, such as processing speed, inhibition, etc., in addition to over composite scores. Though all four sets of brain-environmental associations are valid and important, given our research interest, we focus our discussion on the associations among brain and environmental latent variables from the 2nd set of canonical variables.

To improve the interpretation of how the original observed features contribute to the latent variables, we presented the canonical weights assigned to each variable and the percentage of variance explained. The magnitude of the weight indicates the degree of contribution of the feature to the latent variable. Features with positive weights contribute to the canonical variable positively, while features with negative weights contribute inversely. However, interpreting the importance of a feature based on its canonical weight is subject to the limitation of the beta weights in regression analyses. Small weight can either suggest that the corresponding feature is nonessential, or its contribution has been partially explained by other features due to high multicollinearity. This limitation applies only to the interpretation of the CCA components, not to their extraction since multicollinearity is not a concern for extracting CCA components. CCA is designed to extract multivariate linearly related patterns within each dataset, ensuring that these linear patterns are correlated across datasets. To address the limitation inherent in canonical weights, we also examined the percentage of variance shared which represents the degree to which the observed feature shares variance with the canonical variable.

The accelerated brain age group displayed higher values across all three canonical variables of brain measures compared to the delayed group. First, the ICA gray matter density of the top regions contributed highly to the ICA latent variable. Notably, the first and last contributing regions exhibited positive canonical weights, while the remaining three areas had negative canonical weights (refer to Table 4). Positive weights indicated increased gray matter density in sub-gyral and superior temporal gyrus regions, whereas negative weights indicated reduced gray matter density in middle temporal gyrus and precuneus regions, resulting in higher values of the canonical variable. The sub-gyral region stood out as the most prominent contributor, accounting for 40.58% of the shared variance, followed by the middle temporal gyrus with 38.15%. Therefore, reduced gray matter density in the middle temporal gyrus and precuneus, along with increased density in the superior temporal region, albeit to a lesser extent, contributed to elevated values in the latent gray matter variable. The accelerated group exhibited higher values than the delayed group, implying potentially greater reductions in gray matter density overall, consistent with advanced brain maturation observed during this stage of adolescence (Arain et al., 2013; Giedd et al., 1999; Gogtay and Thompson, 2010; Paus, 2005; Whitford et al., 2007).

Typically, maturation of gray matter volume appears first as linear loss in the dorsal parietal cortices, frontal and occipital poles, then progresses rostrally through the frontal cortex as quadratic and cubic gray matter volume loss (Gogtay et al., 2004). This reduction in gray matter volume is accompanied by increases in cortical thickness in the frontal and parieto-occipital regions as the brain matures (Sowell et al., 2004). Our results are in alignment with the current understanding of the maturation of gray matter volume and cortical thickness. The top five features that contributed to the latent morphological variable were decreases in gray matter volume in the orbitofrontal regions (left medial, and right lateral) and increases in cortical thickness in the right supramarginal region and bilateral occipital regions. The accelerated maturation group demonstrated higher values in this latent variable, confirming that reduction in gray matter volume in the orbitofrontal cortex, alongside increases in cortical thickness in the supramarginal and lateral occipital cortex are associated with the accelerated brain age.

Maturation of functional connectivity during adolescence is marked by increased segregation between short-range connectivity as well as increased integration of long-range connectivity (Fair et al., 2007). This is seen in decreases in connectivity between the anterior PFC, dorsolateral PFC, and the frontoparietal control network in conjunction with increases in connectivity between the dorsal ACC, the medial superior frontal cortex, and the cingula-opercular control network (Stevens et al., 2009). Consistent with this understanding of adolescent maturation, our study showed positive contributions to the latent variable from the functional connectivity of the occipital regions (cuneus, precuneus, and lingual gyrus), while connectivity between the middle temporal gyrus and right inferior frontal gyrus contributed negatively. The accelerated maturation group again had higher values with regard to the latent variable, confirming that they are experiencing accelerated brain maturation.

All three latent brain variables in our study were highly correlated, confirming our expectation that changes in gray matter density, cortical thickness, and functional connectivity are tightly coupled and covary during brain development. Figure 2 also illustrates anatomical regions from the three latent variables that are concentrated in the posterior part of the brain (occipital, temporal, and parietal cortices), accompanied by the inferior orbital part of the frontal cortex. Our results suggest the accelerated brain age group may be experiencing accelerated gray matter density reduction in the middle temporal gyrus and precuneus, volume reduction in the orbitofrontal cortex, as well as increased thickness in the supramarginal region, and increased functional connectivity across cortical-subcortical regions.

The environmental variable linked with the second set of brain patterns reveals that air pollution has the highest shared variance percentage at 84.59% and a canonical weight of −0.26, positioning it as the primary contributing feature (see Table 4). Following air pollution, area crime, and population density emerge as significant contributors to the environmental canonical variable, with canonical weights of 0.06 and 0.01, and shared variance percentages of 16.29 and 8.50%, respectively. Low levels of air pollution coupled with higher area crime and population density result in a heightened latent variable that exhibits positive associations with all three latent brain variables. Although there are no discernible group differences in the environmental variable, its positive correlations with brain variables suggest that exposure to an environment characterized by low air pollution and elevated area crime and population density is associated with patterns of brain regions that include reductions in gray matter and enhanced functional connectivity, regions which are more prominent in the accelerated brain aging group. It seems counterintuitive that lower air pollution, a desirable environmental factor, would be coupled with unfavorable or harsh neighborhood conditions like higher crime rate and population density. However, in terms of brain development/maturation both favorable and unfavorable factors can work in the same direction. Prior research has suggested that individuals may mature faster when exposed to harsh social environmental conditions (Hedderich et al., 2021), and brain development trajectory may be altered by air pollution in a time-sensitive manner (Herting et al., 2024). The lack of group difference in environmental variables could be interpreted in two ways. One is that environmental variables stimulate brain development in specific patterns but with small effect sizes, so large samples are needed to verify environmental effects. The other is that environmental variables are only associated with the part of the variance in the brain patterns not showing brain development differences, and the part showing brain development difference is contributed by something else, such that more investigations on other environmental factors are warranted. In the literature air pollution (specifically fine particulate PM2.5) has been reported to have a positive correlation with gray matter volumetric changes across various brain regions, such as the medial orbitofrontal cortex, while a negative association between air pollution and gray matter volume is observed in regions like the superior temporal gyrus (Miller et al., 2022). Our result showed that a low level of ambient air pollution is linked to a reduction of gray matter density in the middle temporal gyrus and the precuneus, as well as a reduction of gray matter volume in the left medial orbitofrontal cortex and the right lateral orbitofrontal cortex. Moreover, low air pollution is associated with increased gray matter density in the sub-gyral, superior temporal gyrus, and increased cortical thickness in the right supramarginal, bilateral occipital brain regions.

The limitations of our study are also opportunities for future research. Even though our study shows significant associations between environmental factors and brain maturation, we note that these results are only observational. These findings could be affected by potential confounders such as genetics, culture, or other unexplored demographic elements. The mCCA method assumes a linear relationship between observed features and the latent variables, as well as a linear correlation between different datasets. The method will fail to capture hidden relationships if they are complex nonlinear interactions. All our findings are based on ABCD baseline data with a cross-sectional analytical design. A longitudinal analysis of the ABCD cohort that tracks the changes in the multivariate relationships between brain-environment will paint a more complete and likely more complex picture.

In sum, our research leveraged brain age estimation in a large developmental cohort and revealed neuronal structural and functional variations associated with accelerated vs. delayed brain maturation. Furthermore, we provided evidence of associations between environmental factors and brain maturation, suggesting that such factors may modulate neuronal variations. However, the influence of other confounders should be considered. Future longitudinal studies on brain development could further unveil the dynamic trajectory of environmental factors.

## Data Availability

The data analyzed in this study is subject to the following licenses/restrictions: data used in the preparation of this manuscript were obtained from the Adolescent Brain Cognitive Development (ABCD) Study (https://abcdstudy.org) shared by National Institute of Mental Health Data Archive (NDA) (https://nda.nih.gov/). Requests to access these datasets should be directed to https://abcdstudy.org and https://nda.nih.gov/.

## References

[B1] Akshoomoff N. Beaumont J. L. Bauer P. J. Dikmen S. S. Gershon R. C. Mungas D.. (2013). VIII. NIH toolbox cognition battery (CB): composite scores of crystallized, fluid, and overall cognition. Monogr. Soc. Res. Child Dev. 78, 119–132. 10.1111/mono.12038 23952206 PMC4103789

[B2] Alderton A. Villanueva K. O'Connor M. Boulangé C. Badland H. (2019). Reducing inequities in early childhood mental health: How might the neighborhood built environment help close the gap? A systematic search and critical review. Int. J. Environ. Res. Public Health 16:1516. 10.3390/ijerph16091516 31035699 PMC6540328

[B3] Amari S.-I. (1998). Natural gradient works efficiently in learning. Neural Comput. 10, 251–276. 10.1162/089976698300017746

[B4] Arain M. Haque M. Johal L. Mathur P. Nel W. Rais A.. (2013). Maturation of the adolescent brain. Neuropsychiatr. Dis. Treat. 9, 449–461. 10.2147/NDT.S39776 23579318 PMC3621648

[B5] Ashburner J. Barnes G. Chen C.-C. Daunizeau J. Flandin G. Friston K.. (2014). SPM12 Manual. London: Wellcome Trust Centre for Neuroimaging.

[B6] Baecker L. Garcia-Dias R. Vieira S. Scarpazza C. Mechelli A. (2021). Machine learning for brain age prediction: Introduction to methods and clinical applications. EBioMedicine 72:103600. 10.1016/j.ebiom.2021.103600 34614461 PMC8498228

[B7] Baranyi G. Di Marco M. H. Russ T. C. Dibben C. Pearce J. (2021). The impact of neighbourhood crime on mental health: a systematic review and meta-analysis. Soc. Sci. Med. 282:114106. 10.1016/j.socscimed.2021.114106 34139480

[B8] Barnett A. Zhang C. J. Johnston J. M. Cerin E. (2018). Relationships between the neighborhood environment and depression in older adults: a systematic review and meta-analysis. Int. Psychogeriatr. 30, 1153–1176. 10.1017/S104161021700271X 29223174

[B9] Basodi S. Raja R. Ray B. Gazula H. Liu J. Verner E.. (2021). Federation of brain age estimation in structural neuroimaging data. Annu. Int. Conf. IEEE Eng. Med. Biol. Soc. 2021, 3854–3857. 10.1109/EMBC46164.2021.9629865 34892075

[B10] Basodi S. Raja R. Ray B. Gazula H. Sarwate A. D. Plis S.. (2022). Decentralized brain age estimation using mri data. Neuroinformatics 20, 981–990. 10.1007/s12021-022-09570-x 35380365

[B11] Bell A. J. Sejnowski T. J. (1995). An information-maximization approach to blind separation and blind deconvolution. Neural Comput. 7, 1129–1159. 10.1162/neco.1995.7.6.1129 7584893

[B12] Blakemore S. J. Choudhury S. (2006). Development of the adolescent brain: implications for executive function and social cognition. J. Child Psychol. Psychiatry 47, 296–312. 10.1111/j.1469-7610.2006.01611.x 16492261

[B13] Brieant A. E. Sisk L. M. Gee D. G. (2021). Associations among negative life events, changes in cortico-limbic connectivity, and psychopathology in the abcd study. Dev. Cogn. Neurosci. 52:101022. 10.1016/j.dcn.2021.101022 34710799 PMC8556598

[B14] Bush N. R. Wakschlag L. S. LeWinn K. Z. Hertz-Picciotto I. Nozadi S. S. Pieper S.. (2020). Family environment, neurodevelopmental risk, and the environmental influences on child health outcomes (echo) initiative: looking back and moving forward. Front. Psychiatry 11:547. 10.3389/fpsyt.2020.00547 32636769 PMC7318113

[B15] Cairns J.-M. Graham E. Bambra C. (2017). Area-level socioeconomic disadvantage and suicidal behaviour in europe: a systematic review. Soc. Sci. Med. 192, 102–111. 10.1016/j.socscimed.2017.09.034 28965001

[B16] Casanova R. Anderson A. Barnard R. Walker K. Hughes T. Kritchevsky S.. (2022). Accelerated brain aging is associated with mortality across race. Innovat. Aging 6(Suppl. 1):784. 10.1093/geroni/igac059.2834

[B17] Casey B. Tottenham N. Liston C. Durston S. (2005). Imaging the developing brain: what have we learned about cognitive development? Trends Cogn. Sci. 9, 104–110. 10.1016/j.tics.2005.01.011 15737818

[B18] Chapman J. Wang H.-T. (2021). CCA-zoo: a collection of regularized, deep learning based, kernel, and probabilistic cca methods in a scikit-learn style framework. J. Open Source Softw. 6:3823. 10.21105/joss.03823

[B19] Cipriani G. Danti S. Carlesi C. Borin G. (2018). Danger in the air: air pollution and cognitive dysfunction. Am. J. Alzheimers Dis. Other Dement. 33, 333–341. 10.1177/1533317518777859 29874918 PMC10852418

[B20] Correa N. Adali T. Li Y.-O. Calhoun V. D. (2005). “Comparison of blind source separation algorithms for fMRI using a new matlab toolbox: gift,” in Proceedings.(ICASSP'05). IEEE International Conference on Acoustics, Speech, and Signal Processing, 2005, Vol. 5 (Philadelphia, PA: IEEE), v-401.

[B21] Desikan R. S. Ségonne F. Fischl B. Quinn B. T. Dickerson B. C. Blacker D.. (2006). An automated labeling system for subdividing the human cerebral cortex on mri scans into gyral based regions of interest. Neuroimage 31, 968–980. 10.1016/j.neuroimage.2006.01.021 16530430

[B22] Dimitrov-Discher A. Wenzel J. Kabisch N. Hemmerling J. Bunz M. Schöndorf J.. (2022). Residential green space and air pollution are associated with brain activation in a social-stress paradigm. Sci. Rep. 12:10614. 10.1038/s41598-022-14659-z 35739150 PMC9226020

[B23] Dosenbach N. U. Nardos B. Cohen A. L. Fair D. A. Power J. D. Church J. A.. (2010). Prediction of individual brain maturity using fmri. Science 329, 1358–1361. 10.1126/science.1194144 20829489 PMC3135376

[B24] Du Y. Fu Z. Sui J. Gao S. Xing Y. Lin D.. (2020). Neuromark: An automated and adaptive ica based pipeline to identify reproducible fmri markers of brain disorders. Neuroimage Clin. 28:102375. 10.1016/j.nicl.2020.102375 32961402 PMC7509081

[B25] Dunlop K. Victoria L. W. Downar J. Gunning F. M. Liston C. (2021). Accelerated brain aging predicts impulsivity and symptom severity in depression. Neuropsychopharmacology 46, 911–919. 10.1038/s41386-021-00967-x 33495545 PMC8115107

[B26] Fair D. A. Dosenbach N. U. Church J. A. Cohen A. L. Brahmbhatt S. Miezin F. M.. (2007). Development of distinct control networks through segregation and integration. Proc. Nat. Acad. Sci. U. S. A. 104, 13507–13512. 10.1073/pnas.0705843104 17679691 PMC1940033

[B27] Franke K. Gaser C. (2019). Ten years of brainage as a neuroimaging biomarker of brain aging: what insights have we gained? Front. Neurol. 10:789. 10.3389/fneur.2019.00789 31474922 PMC6702897

[B28] Franke K. Ziegler G. Klöppel S. Gaser C. Initiative A. D. N.. (2010). Estimating the age of healthy subjects from t1-weighted mri scans using kernel methods: exploring the influence of various parameters. Neuroimage 50, 883–892. 10.1016/j.neuroimage.2010.01.005 20070949

[B29] Fu Z. Liu J. Salman M. S. Sui J. Calhoun V. D. (2023). Functional connectivity uniqueness and variability? Linkages with cognitive and psychiatric problems in children. Nat. Mental Health 1, 956–970. 10.1038/s44220-023-00151-8

[B30] Gapp K. Woldemichael B. T. Bohacek J. Mansuy I. (2014). Epigenetic regulation in neurodevelopment and neurodegenerative diseases. Neuroscience 264, 99–111. 10.1016/j.neuroscience.2012.11.040 23256926

[B31] Giedd J. N. Blumenthal J. Jeffries N. O. Castellanos F. X. Liu H. Zijdenbos A.. (1999). Brain development during childhood and adolescence: a longitudinal MRI study. Nat. Neurosci. 2, 861–863. 10.1038/13158 10491603

[B32] Gogtay N. Giedd J. N. Lusk L. Hayashi K. M. Greenstein D. Vaituzis A. C.. (2004). Dynamic mapping of human cortical development during childhood through early adulthood. Proc. Nat. Acad. Sci. U. S. A. 101, 8174–8179. 10.1073/pnas.0402680101 15148381 PMC419576

[B33] Gogtay N. Thompson P. M. (2010). Mapping gray matter development: implications for typical development and vulnerability to psychopathology. Brain Cogn. 72, 6–15. 10.1016/j.bandc.2009.08.009 19796863 PMC2815268

[B34] Green J. G. McLaughlin K. A. Berglund P. A. Gruber M. J. Sampson N. A. Zaslavsky A. M.. (2010). Childhood adversities and adult psychiatric disorders in the national comorbidity survey replication I: associations with first onset of DSM-IV disorders. Arch. Gen. Psychiatry 67, 113–123. 10.1001/archgenpsychiatry.2009.186 20124111 PMC2822662

[B35] Guxens M. Lubczyńska M. J. Muetzel R. L. Dalmau-Bueno A. Jaddoe V. W. Hoek G.. (2018). Air pollution exposure during fetal life, brain morphology, and cognitive function in school-age children. Biol. Psychiatry 84, 295–303. 10.1016/j.biopsych.2018.01.016 29530279

[B36] Hackman D. A. Cserbik D. Chen J. C. Berhane K. Minaravesh B. McConnell R.. (2021). Association of local variation in neighborhood disadvantage in metropolitan areas with youth neurocognition and brain structure. JAMA Pediatr. 175, e210426. 10.1001/jamapediatrics.2021.0426 33938908 PMC8094040

[B37] Hagler D. J. Hatton S. Cornejo M. D. Makowski C. Fair D. A. Dick A. S.. (2019). Image processing and analysis methods for the adolescent brain cognitive development study. Neuroimage 202:116091. 10.1016/j.neuroimage.2019.116091 31415884 PMC6981278

[B38] Hardoon D. R. Szedmak S. Shawe-Taylor J. (2004). Canonical correlation analysis: an overview with application to learning methods. Neural Comput. 16, 2639–2664. 10.1162/0899766042321814 15516276

[B39] Hedderich D. M. Menegaux A. Schmitz-Koep B. Nuttall R. Zimmermann J. Schneider S. C.. (2021). Increased brain age gap estimate (brainage) in young adults after premature birth. Front. Aging Neurosci. 13:653365. 10.3389/fnagi.2021.653365 33867970 PMC8047054

[B40] Herting M. M. Bottenhorn K. L. Cotter D. L. (2024). Outdoor air pollution and brain development in childhood and adolescence. Trends Neurosci. 47, 593–607. 10.1016/j.tins.2024.06.008 39054161 PMC11324378

[B41] Herzberg M. P. Gunnar M. R. (2020). Early life stress and brain function: activity and connectivity associated with processing emotion and reward. Neuroimage 209:116493. 10.1016/j.neuroimage.2019.116493 31884055 PMC7056544

[B42] Holmes C. Levy M. Smith A. Pinne S. Neese P. (2015). A model for creating a supportive trauma-informed culture for children in preschool settings. J. Child Fam. Stud. 24, 1650–1659. 10.1007/s10826-014-9968-6 25972726 PMC4419190

[B43] Huttenlocher P. R. Dabholkar A. S. (1997). Regional differences in synaptogenesis in human cerebral cortex. J. Comp. Neurol. 387, 167–178. 10.1002/(SICI)1096-9861(19971020)387:2<167::AID-CNE1>3.0.CO;2-Z 9336221

[B44] Jenkinson M. Beckmann C. F. Behrens T. E. Woolrich M. W. Smith S. M. (2012). FSL. Neuroimage 62, 782–790. 10.1016/j.neuroimage.2011.09.015 21979382

[B45] Jeong H. J. Moore T. M. Durham E. L. Reimann G. E. Dupont R. M. Cardenas-Iniguez C.. (2023). General and specific factors of environmental stress and their associations with brain structure and dimensions of psychopathology. Biol. Psychiatry Glob. Open Sci. 3, 480–489. 10.1016/j.bpsgos.2022.04.004 37519461 PMC10382692

[B46] Jorgensen N. A. Muscatell K. A. McCormick E. M. Prinstein M. J. Lindquist K. A. Telzer E. H. (2023). Neighborhood disadvantage, race/ethnicity and neural sensitivity to social threat and reward among adolescents. Soc. Cogn. Affect. Neurosci. 18:nsac053. 10.1093/scan/nsac053 36178870 PMC9949505

[B47] Kaufmann T. Alnaes D. Doan N. T. Brandt C. L. Andreassen O. A. Westlye L. T. (2017). Delayed stabilization and individualization in connectome development are related to psychiatric disorders. Nat. Neurosci. 20, 513–515. 10.1038/nn.4511 28218917

[B48] Khan A. R. Wang L. Beg M. F. (2008). Freesurfer-initiated fully-automated subcortical brain segmentation in mri using large deformation diffeomorphic metric mapping. Neuroimage 41, 735–746. 10.1016/j.neuroimage.2008.03.024 18455931 PMC2905149

[B49] Konrad K. Firk C. Uhlhaas P. J. (2013). Brain development during adolescence: neuroscientific insights into this developmental period. Dtsch. Arztebl. Int. 110, 425–431. 10.3238/arztebl.2013.0425 23840287 PMC3705203

[B50] Kwon H. Reiss A. L. Menon V. (2002). Neural basis of protracted developmental changes in visuo-spatial working memory. Proc. Natl. Acad. Sci. U. S. A. 99, 13336–13341. 10.1073/pnas.162486399 12244209 PMC130634

[B51] Laruelle M. Kegeles L. Zea-Ponce Y. Mawlawi O. Martinez D. Abi-Dargham A.. (2002). Amphetamine-induced dopamine release in patients with schizotypal personality disorders studied by spect and [1231] ibzm. Neuroimage 16, S61–S61.15121484

[B52] Lederbogen F. Haddad L. Meyer-Lindenberg A. (2013). Urban social stress-risk factor for mental disorders. The case of schizophrenia. Environ. Pollut. 183, 2–6. 10.1016/j.envpol.2013.05.046 23791151

[B53] Li Y. O. Adali T. Wang W. Calhoun V. D. (2009). Joint blind source separation by multi-set canonical correlation analysis. IEEE Trans. Signal Process. 57, 3918–3929. 10.1109/TSP.2009.2021636 20221319 PMC2835373

[B54] Liem F. Varoquaux G. Kynast J. Beyer F. Kharabian Masouleh S. Huntenburg J. M.. (2017). Predicting brain-age from multimodal imaging data captures cognitive impairment. Neuroimage 148, 179–188. 10.1016/j.neuroimage.2016.11.005 27890805

[B55] Luo N. Sui J. Abrol A. Chen J. Turner J. A. Damaraju E.. (2020). Structural brain architectures match intrinsic functional networks and vary across domains: a study from 15 000+ individuals. Cereb. Cortex 30, 5460–5470. 10.1093/cercor/bhaa127 32488253 PMC7566687

[B56] Miller D. J. Duka T. Stimpson C. D. Schapiro S. J. Baze W. B. McArthur M. J.. (2012). Prolonged myelination in human neocortical evolution. Proc. Natl. Acad. Sci. U. S. A. 109, 16480–16485. 10.1073/pnas.1117943109 23012402 PMC3478650

[B57] Miller J. G. Dennis E. L. Heft-Neal S. Jo B. Gotlib I. H. (2022). Fine particulate air pollution, early life stress, and their interactive effects on adolescent structural brain development: a longitudinal tensor-based morphometry study. Cereb. Cortex 32, 2156–2169. 10.1093/cercor/bhab346 34607342 PMC9113318

[B58] Modabbernia A. Janiri D. Doucet G. E. Reichenberg A. Frangou S. (2021). Multivariate patterns of brain-behavior-environment associations in the adolescent brain and cognitive development study. Biol. Psychiatry 89, 510–520. 10.1016/j.biopsych.2020.08.014 33109338 PMC7867576

[B59] Paus T. (2005). Mapping brain maturation and cognitive development during adolescence. Trends Cogn. Sci. 9, 60–68. 10.1016/j.tics.2004.12.008 15668098

[B60] Paus T. Zijdenbos A. Worsley K. Collins D. L. Blumenthal J. Giedd J. N.. (1999). Structural maturation of neural pathways in children and adolescents: in vivo study. Science 283, 1908–1911. 10.1126/science.283.5409.1908 10082463

[B61] Peng H. Gong W. Beckmann C. F. Vedaldi A. Smith S. M. (2021). Accurate brain age prediction with lightweight deep neural networks. Med. Image Anal. 68:101871. 10.1016/j.media.2020.101871 33197716 PMC7610710

[B62] Phillips N. S. Baedke J. L. Williams A. Ji Q. Zhang S. Scoggins M.. (2023). Accelerated brain age and associated neurocognitive impairments in adult survivors of childhood cancer. J. Clin. Oncol. 41(16_suppl), 10028–10028. 10.1200/JCO.2023.41.16_suppl.10028

[B63] Pujol J. Martínez-Vilavella G. Macià D. Fenoll R. Alvarez-Pedrerol M. Rivas I.. (2016). Traffic pollution exposure is associated with altered brain connectivity in school children. Neuroimage 129, 175–184. 10.1016/j.neuroimage.2016.01.036 26825441

[B64] Rachakonda S. Egolf E. Correa N. Calhoun V. (2007). Group ICA of fMRI toolbox (gift) manual. Dostupnez.24120443

[B65] Rakesh D. Zalesky A. Whittle S. (2021). Similar but distinct - effects of different socioeconomic indicators on resting state functional connectivity: findings from the adolescent brain cognitive development (abcd) study(r). Dev. Cogn. Neurosci. 51:101005. 10.1016/j.dcn.2021.101005 34419766 PMC8379618

[B66] Rakesh D. Zalesky A. Whittle S. (2022). Assessment of parent income and education, neighborhood disadvantage, and child brain structure. JAMA Netw. Open 5:e2226208. 10.1001/jamanetworkopen.2022.26208 35980639 PMC9389347

[B67] Rakesh D. Zalesky A. Whittle S. (2023). The role of school environment in brain structure, connectivity, and mental health in children: a multimodal investigation. Biol. Psychiatry Cogn. Neurosci. Neuroimaging 8, 32–41. 10.1016/j.bpsc.2022.01.006 35123109

[B68] Ramduny J. Bastiani M. Huedepohl R. Sotiropoulos S. N. Chechlacz M. (2022). The association between inadequate sleep and accelerated brain ageing. Neurobiol. Aging 114, 1–14. 10.1016/j.neurobiolaging.2022.02.005 35344818 PMC9084918

[B69] Ray B. Chen J. Fu Z. Suresh P. Thapaliya B. Farahdel B.. (2023). Replication and refinement of brain age model for adolescent development. bioRxiv. 10.1101/2023.08.16.553472 37645839 PMC10462059

[B70] Ray B. Chen J. Fu Z. Suresh P. Thapaliya B. Farahdel B.. (2024). “Replication and refinement of brain age model for adolescent development,” in 2024 IEEE International Symposium on Biomedical Imaging (ISBI) (Athens: IEEE), 1–5. 10.1109/ISBI56570.2024.10635532

[B71] Ray B. Duan K. Chen J. Fu Z. Suresh P. Johnson S.. (2021). Multimodal brain age prediction with feature selection and comparison. Annu. Int. Conf. IEEE Eng. Med. Biol. Soc. 2021, 3858–3864. 10.1109/EMBC46164.2021.9631007 34892076

[B72] Rubia K. Overmeyer S. Taylor E. Brammer M. Williams S. C. Simmons A.. (2000). Functional frontalisation with age: mapping neurodevelopmental trajectories with fMRI. Neurosci. Biobehav. Rev. 24, 13–19. 10.1016/S0149-7634(99)00055-X 10654655

[B73] Saha R. Saha D. K. Rahaman M. A. Fu Z. Calhoun V. D. (2022). “Longitudinal whole-brain functional network change patterns over a two-year period in the abcd data,” in 2022 IEEE 19th International Symposium on Biomedical Imaging (ISBI) (Kolkata: IEEE), 1–4. 10.1109/ISBI52829.2022.9761647

[B74] Sampson L. Ettman C. K. Galea S. (2020). Urbanization, urbanicity, and depression: a review of the recent global literature. Curr. Opin. Psychiatry 33, 233–244. 10.1097/YCO.0000000000000588 32040041

[B75] Satterthwaite T. D. Connolly J. J. Ruparel K. Calkins M. E. Jackson C. Elliott M. A.. (2016). The philadelphia neurodevelopmental cohort: a publicly available resource for the study of normal and abnormal brain development in youth. Neuroimage 124(Pt B), 1115–1119. 10.1016/j.neuroimage.2015.03.056 25840117 PMC4591095

[B76] Smith K. E. Pollak S. D. (2020). Early life stress and development: potential mechanisms for adverse outcomes. J. Neurodev. Disord. 12, 1–15. 10.1186/s11689-020-09337-y 33327939 PMC7745388

[B77] Smith S. M. Jenkinson M. Woolrich M. W. Beckmann C. F. Behrens T. E. Johansen-Berg H.. (2004). Advances in functional and structural mr image analysis and implementation as fsl. Neuroimage 23(Suppl. 1), S208–S219. 10.1016/j.neuroimage.2004.07.051 15501092

[B78] Sowell E. R. Thompson P. M. Leonard C. M. Welcome S. E. Kan E. Toga A. W. (2004). Longitudinal mapping of cortical thickness and brain growth in normal children. J. Neurosci. 24, 8223–8231. 10.1523/JNEUROSCI.1798-04.2004 15385605 PMC6729679

[B79] Sowell E. R. Thompson P. M. Tessner K. D. Toga A. W. (2001). Mapping continued brain growth and gray matter density reduction in dorsal frontal cortex: inverse relationships during postadolescent brain maturation. J. Neurosci. 21, 8819–8829. 10.1523/JNEUROSCI.21-22-08819.2001 11698594 PMC6762261

[B80] Stevens M. C. Pearlson G. D. Calhoun V. D. (2009). Changes in the interaction of resting-state neural networks from adolescence to adulthood. Hum. Brain Mapp. 30, 2356–2366. 10.1002/hbm.20673 19172655 PMC6788906

[B81] Sui Y. Ettema D. Helbich M. (2022). Longitudinal associations between the neighborhood social, natural, and built environment and mental health: A systematic review with meta-analyses. Health Place 77:102893. 10.1016/j.healthplace.2022.102893 35988452

[B82] Taylor R. L. Cooper S. R. Jackson J. J. Barch D. M. (2020). Assessment of neighborhood poverty, cognitive function, and prefrontal and hippocampal volumes in children. JAMA Netw. Open 3:e2023774. 10.1001/jamanetworkopen.2020.23774 33141160 PMC7610187

[B83] Thapaliya B. Calhoun V. D. Liu J. (2021). “Environmental and genome-wide association study on children anxiety and depression,” in 2021 IEEE International Conference on Bioinformatics and Biomedicine (BIBM) (Houston, TX: IEEE), 2330–2337. 10.1109/BIBM52615.2021.9669291

[B84] Thapaliya B. Ray B. Farahdel B. Suresh P. Sapkota R. consortium I.. (2023). Cross-continental environmental and genome-wide association study on children and adolescent anxiety and depression. medRxiv. 10.21203/rs.3.rs-2744140/v1 38827440 PMC11141390

[B85] Thapaliya B. Ray B. Farahdel B. Suresh P. Sapkota R. Holla B.. (2024). Cross-continental environmental and genome-wide association study on children and adolescent anxiety and depression. Front. Psychiatry 15:1384298. 10.3389/fpsyt.2024.1384298 38827440 PMC11141390

[B86] Tomasi D. Volkow N. D. (2021). Associations of family income with cognition and brain structure in usa children: prevention implications. Mol. Psychiatry 26, 6619–6629. 10.1038/s41380-021-01130-0 33990770 PMC8590701

[B87] Tomasi D. Volkow N. D. (2023). Effects of family income on brain functional connectivity in us children: associations with cognition. Mol. Psychiatry 28, 4195–4202. 10.1038/s41380-023-02222-9 37580525

[B88] Van Essen D. C. Ugurbil K. Auerbach E. Barch D. Behrens T. E. Bucholz R.. (2012). The human connectome project: a data acquisition perspective. Neuroimage 62, 2222–2231. 10.1016/j.neuroimage.2012.02.018 22366334 PMC3606888

[B89] Whitford T. J. Rennie C. J. Grieve S. M. Clark C. R. Gordon E. Williams L. M. (2007). Brain maturation in adolescence: concurrent changes in neuroanatomy and neurophysiology. Hum. Brain Mapp. 28, 228–237. 10.1002/hbm.20273 16767769 PMC6871488

[B90] Woolrich M. W. Jbabdi S. Patenaude B. Chappell M. Makni S. Behrens T.. (2009). Bayesian analysis of neuroimaging data in fsl. Neuroimage 45(1 Suppl), S173–S186. 10.1016/j.neuroimage.2008.10.055 19059349

[B91] Xu J. Liu N. Polemiti E. Garcia-Mondragon L. Tang J. Liu X.. (2023). Effects of urban living environments on mental health in adults. Nat. Med. 29, 1456–1467. 10.1038/s41591-023-02365-w 37322117 PMC10287556

[B92] Xu J. Liu X. Li Q. Goldblatt R. Qin W. Liu F.. (2022). Global urbanicity is associated with brain and behaviour in young people. Nat. Hum. Behav. 6, 279–293. 10.1038/s41562-021-01204-7 34711977

[B93] Xu L. Groth K. M. Pearlson G. Schretlen D. J. Calhoun V. D. (2009). Source-based morphometry: the use of independent component analysis to identify gray matter differences with application to schizophrenia. Hum. Brain Mapp. 30, 711–724. 10.1002/hbm.20540 18266214 PMC2751641

[B94] Zhuang X. Yang Z. Cordes D. (2020). A technical review of canonical correlation analysis for neuroscience applications. Hum. Brain Mapp. 41, 3807–3833. 10.1002/hbm.25090 32592530 PMC7416047

