# Supplementary material for: Adolescent brain maturation associated with environmental factors: a multivariate analysis
Source: Front Neuroimaging. 2024 Nov 19;3:1390409. doi: 10.3389/fnimg.2024.1390409 (PMC11613425; doi:10.3389/fnimg.2024.1390409)
Supplement: Supplementary file 1 [file Data_Sheet_1.pdf]

Supplementary Material

Adolescent Brain Maturation Associated with Environmental Factors: A Multivariate Analysis

Bhaskar Ray, Dwan Jensen, Pranav Suresh, Bishal Thapaliya, Ram Sapkota, Britny Farahdel, Zening Fu, Jiayu Chen, Vince D. Calhoun, Jingyu Liu*

*** Correspondence:** Jingyu Liu: jliu75@gsu.edu

1 Supplementary Data: Environmental Factors

A detailed explanation of nine environmental factors is presented below.

1) Air Pollution: (Higher- Bad, Lower- Good)

- 3 years average of ground level NO2.
- Annual average of PM 2.5 at primary residential address.

2) Population Density: (Higher- Bad, Lower- Good)

- Unadjusted population density
- Gross residential density

3) Area Crime: (Higher- Bad, Lower- Good)

- Uniform Crime Reports: total adult offenses
- Uniform Crime Reports: drug sale total
- Uniform Crime Reports: drug possession total
- Uniform Crime Reports: drive under influence

4) Neighborhood Safety: (Higher- Good, Lower- Bad)

- I feel safe walking in my neighborhood, day or night.
- Violence is not a problem in my neighborhood.
- My neighborhood is safe from crime.

5) School Safety: (Higher- Good, Lower- Bad)

- Students have lots of chances to help decide things like class activities and rules.

- I get along with my teachers.
- I feel safe at my school.
- My teacher(s) notices when I am doing a good job and lets me know.

6) Household Income:

- Income [0, 1= Less than $5,000; 2=$5,000 through $11,999; 3=$12,000 through $15,999; 4=$16,000 through $24,999; 5=$25,000 through $34,999; 6=$35,000 through $49,999; 7=$50,000 through $74,999; 8=$75,000 through $99,999; 9=$100,000 through $199,999; 10=$200,000 and greater]

7) Family Conflict: (Higher- Bad, Lower- Good) – Some variables are reversed to get the sum

- We fight a lot in our family.
- Family members rarely become openly angry.
- Family members sometimes get so angry they throw things.
- Family members hardly ever lose their tempers.
- Family members often criticize each other.
- Family members sometimes hit each other.
- If there's a disagreement in our family, we try hard to smooth things over and keep the peace.
- Family members often try to one-up or outdo each other.
- In our family, we believe you do not ever get anywhere by raising your voice.

9) Area Deprivation**:** (Higher- Bad, Lower- Good) – Some variables were reversed to get the sum

- Percentage of population aged >=25 y with at least a high school diploma.

- Median family income.
- Percentage of civilian labor force population aged >=16 y unemployed (unemployment rate).
- Percentage of families below the poverty level.
- Percentage of population below 138% of the poverty threshold.

9) Early Life Stress: (Higher- Bad, Lower- Good) – Some variables were reversed to get the sum

Six categories were taken in consideration including Physical abuse/Expose or experience to trauma/Sexual Abuse; Household substance abuse; Household mental illness; Criminal in household; Parent separation /divorce; Emotional Neglect.

2 Supplementary Figure

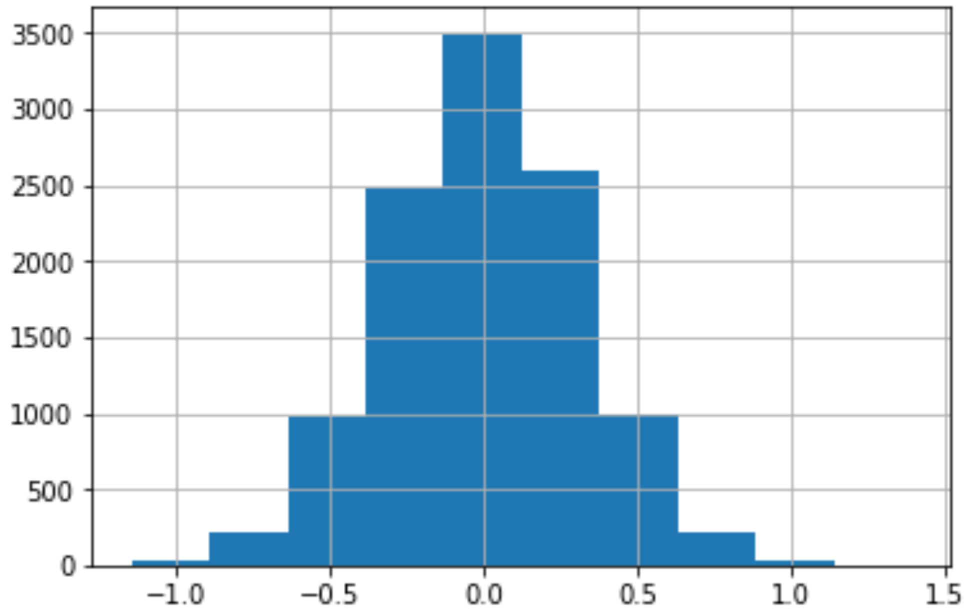

Supplementary Figure 1: Plot of brain age gaps (estimated brain age - chronicle age) of ABCD baseline subjects from refined brain age model.

3. To provide more transparent and comprehensive view of our findings we have included the data for all four sets of canonical variables from the training and testing set in this section.

Supplementary Table 1: Top five contributing features of the 1st set of canonical variables and their canonical weights and shared variance percentage in the training data.

Canonical Variate	Top 5 Contributing Features	Canonical Weights	Shared Variance Percentage (r2%)
**ICA GM**	Declive	0.09	53.27
	Inferior Parietal Lobule	-0.10	45.73
	Middle Occipital Gyrus	0.05	37.03
	Middle Frontal Gyrus	0.07	35.72
	Fusiform Gyrus	-0.04	33.40
**FreeSurfer**	Intra Cranial Volume	0.04	74.96
	Left Lateral Orbitofrontal Cortical Volume	0.03	54.65

	Right Lateral Occipital Cortical Volume	0.06	53.94
	Left Superior Frontal Cortical Volume	0.04	53.25
	Right Fusiform Cortical Volume	0.04	51.77
**sFNC**	Hippocampus, Precuneus	0.03	11.19
	Thalamus, Middle Temporal Gyrus	0.01	9.61
	Thalamus, Fusiform Gyrus	0.015	8.76
	Fusiform Gyrus, Hippocampus	0.03	8.51
	Superior Temporal Gyrus, Anterior Cingulate Cortex	0.01	7.82
**Environmental Factors**	Household Income	0.16	70.87
	Area Deprivation	-0.14	69.52
	Neighborhood Safety	0.06	36.10
	Air Pollution	-0.09	18.33
	ELS	-0.04	12.22

Supplementary Table 2: Top five contributing features of the 1st set of canonical variables and their canonical weights and shared variance percentage in the testing data.

**Canonical Variate**	**Top 5 Contributing Features**	**Canonical Weights**	**Shared Variance Percentage (r2%)**
**ICA GM**	Declive	0.09	52.12
	Inferior Parietal Lobule 1	-0.10	40.77
	Middle Frontal Gyrus	0.07	37.16
	Middle Occipital Gyrus	0.05	33.47
	Posterior Cingulate	0.05	31.33
**FreeSurfer**	Intra Cranial Volume	0.04	72.28

	Left Lateral Orbitofrontal Cortical Volume	0.03	55.95
	Right Lateral Occipital Cortical Volume	0.06	53.11
	Right Fusiform Cortical Volume	0.04	52.36
	Left Precentral Cortical Volume	0.03	50.59
**sFNC**	Thalamus, Middle Temporal Gyrus	0.01	15.72
	Hippocampus, Precuneus	0.03	13.72
	Thalamus, Postcentral Gyrus	0.01	12.30
	Fusiform Gyrus, Hippocampus	0.03	9.99
	Calcarine Gyrus, Hippocampus	0.01	9.56
**Environmental Factors**	Household Income	0.16	71.95
	Area Deprivation	-0.14	68.56
	Neighborhood Safety	0.06	38.31
	Air Pollution	-0.09	17.56
	Population Density	0.01	17.12

Supplementary Table 3: Top five contributing features of the 2nd set of canonical variables and their canonical weights and shared variance percentage in the training data.

**Canonical Variate**	**Top 5 Contributing Features**	**Canonical Weights**	**Shared Variance Percentage (r2%)**
**ICA GM**	Middle Temporal Gyrus 1	-0.06	38.99
	Sub-Gyral 1	0.15	37.63
	Precuneus	-0.009	32.19

	Tuber	-0.001	29.03
	Middle Temporal Gyrus 2	-0.07	26.48
**FreeSurfer**	Left Medial Orbitofrontal Cortical Volume	-0.10	34.50
	Left Lateral Occipital Cortical Thickness	0.09	23.61
	Right Supra Marginal Cortical Thickness	0.05	22.44
	Right Lateral Orbitofrontal Cortical Volume	-0.08	21.85
	Right Lateral Occipital Cortical Thickness	0.07	20.69
**sFNC**	Hippocampus, Precuneus	0.02	14.70
	Thalamus, Precentral Gyrus	0.02	12.76
	Hippocampus, Precuneus	0.015	12.46
	Putamen, Thalamus	0.02	12.25
	Calcarine Gyrus, Hippocampus	0.01	12.02
**Environmental Factors**	Air Pollution	-0.26	84.61
	Area crime	0.06	21.97
	Population Density	0.01	8.92
	Family Conflicts	0.04	1.91
	Neighborhood Safety	0.01	1.56

Supplementary Table 4: Top five contributing features of the 2nd set of canonical variables and their canonical weights and shared variance percentage in the testing data.

**Canonical Variate**	**Top 5 Contributing Features**	**Canonical Weights**	**Shared Variance Percentage (r2%)**
**ICA GM**	Sub-Gyral 1	0.15	40.58
	Middle Temporal Gyrus 1	-0.06	38.15
	Precuneus	-0.01	33.26
	Middle Temporal Gyrus 2	-0.07	27.36
	Superior Temporal Gyrus	0.03	26.69
**FreeSurfer**	Left Medial Orbitofrontal Cortical Volume	-0.10	32.33
	Right Supramarginal Cortical Thickness	0.05	24.30
	Left Lateral Occipital Cortical Thickness	0.09	23.64
	Right Lateral Occipital Cortical Thickness	0.07	22.73
	Right Lateral Orbitofrontal Cortical Volume	-0.08	20.78
**sFNC**	Lingual Gyrus, Precuneus	0.02	15.23
	Middle Temporal Gyrus, Right Inferior Frontal Gyrus	-0.02	13.10
	Cuneus, Hippocampus	0.02	12.84
	Cuneus, Precuneus	0.004	12.25
	Lingual Gyrus, Hippocampus	0.01	12.25
**Environmental Factors**	Air Pollution	-0.26	84.59
	Area Crime	0.06	16.29
	Population Density	0.01	8.50
	Family Conflicts	0.04	1.92
	Neighborhood Safety	0.01	1.01

Supplementary Table 5: Top five contributing features of the 3rd set of canonical variables and their canonical weights and shared variance percentage in the training data.

Canonical Variate	Top 5 Contributing Features	Canonical Weights	Shared Variance Percentage (r2%)
**ICA GM**	Sub-Gyral 2	-0.25	27.70
	Culmen	-0.05	17.95
	Angular Gyrus	-0.10	16.96
	Posterior Cingulate	0.11	13.03
	Sub-Gyral 3	0.06	11.65
**FreeSurfer**	Left Cuneus Cortical Volume	-0.10	21.84
	Left Para Hippocampal Cortical Thickness	-0.11	18.77
	Left Pericalcarine Cortical Volume	-0.06	17.92
	Left Banks of Superior Temporal Sulcus Cortical Volume	0.06	15.35
	Right Cuneus Cortical Volume	-0.08	14.92
**sFNC**	Superior Temporal Gyrus, Middle Temporal Gyrus	0.01	8.68
	Paracentral Lobule, Middle Temporal Gyrus	-0.03	8.55
	Superior Parietal Lobule, Precuneus	0.024	8.17
	Superior Parietal Lobule, Middle Temporal Gyrus	-0.04	7.68
	Middle Temporal Gyrus, Left Inferior Parietal Lobule	-0.02	6.69
**Environmental Factors**	Area Deprivation	0.12	76.63
	Household Income	-0.02	43.20

	Neighborhood Safety	-0.04	38.16
	Population Density	0.03	11.62
	ELS	0.01	11.13

Supplementary Table 6: Top five contributing features of the 3rd set of canonical variables and their canonical weights and shared variance percentage in the testing data.

**Canonical Variate**	**Top 5 Contributing Features**	**Canonical Weights**	**Shared Variance Percentage (r2%)**
**ICA GM**	Sub-Gyral 2	-0.25	29.84
	Culmen	-0.05	17.92
	Angular Gyrus	-0.10	17.51
	Precuneus	0.07	16.61
	Posterior Cingulate	0.11	12.79
**FreeSurfer**	Left Cuneus Cortical Volume	-0.10	26.21
	Left Peri Calcarine Cortical Volume	-0.06	23.14
	Right Cuneus Cortical Volume	-0.08	17.97
	Right Pericalcarine Cortical Volume	-0.05	17.41
	Right Banks of Superior Temporal Sulcus Cortical Volume	0.05	17.18
**sFNC**	Superior Parietal Lobule, Precuneus	0.005	9.41
	Superior Temporal Gyrus, Middle temporal Gyrus	0.01	8.31
	Paracentral Lobule, Middle Temporal Gyrus	-0.03	8.19
	Superior Parietal lobule, Middle Temporal Gyrus	-0.04	7.54
	Superior Parietal Lobule, Precuneus	0.020	6.96

**Environmental Factors**	Area Deprivation	0.12	77.11
	Neighborhood Safety	-0.04	44.95
	Household Income	-0.02	44.15
	Population Density	0.03	15.50
	ELS	0.01	12.76

Supplementary Table 7: Top five contributing features of the 4th set of canonical variables and their canonical weights and shared variance percentage in the training data.

Canonical Variate	Top 5 Contributing Features	Canonical Weights	Shared Variance Percentage (r2%)
**ICA GM**	Angular Gyrus	0.10	13.50
	Inferior Frontal Gyrus	-0.07	9.02
	Sub-Gyral 3	-0.07	8.55
	Inferior Parietal Lobule 2	0.04	8.20
	Middle Temporal Gyrus 2	0.10	7.45
**FreeSurfer**	Right Superior Temporal Cortical Thickness	-0.05	9.43
	Right Postcentral Cortical Thickness	-0.11	9.37
	Right Pars Triangularis Cortical Volume	0.11	8.65
	Left Fusiform Cortical Thickness	-0.02	8.28
	Right Banks of Superior Temporal Sulcus Cortical Thickness	-0.03	7.84
**sFNC**	Postcentral Gyrus, Postcentral Gyrus	0.08	30.68
	Paracentral Lobule, Superior Parietal Lobule	0.02	19.57
	Superior Medial Frontal Gyrus, Inferior Parietal Lobule	-0.02	18.40
	Paracentral Lobule, Inferior Parietal Lobule	0.01	17.57
	Inferior Parietal Lobule, Left Inferior Parietal Lobule	0.01	15.87
**Environmental Factors**	Household Income	-0.10	68.54
	Area Deprivation	0.06	47.32
	School Safety	-0.06	15.45

	Neighborhood Safety	0.004	13.40
	ELS	0.01	8.78

Supplementary Table 8: Top five contributing features of the 4th set of canonical variables and their canonical weights and shared variance percentage in the testing data.

**Canonical Variate**	**Top 5 Contributing Features**	**Canonical Weights**	**Shared Variance Percentage (r2%)**
**ICA GM**	Inferior Frontal Gyrus	-0.07	12.38
	Sub-Gyral 3	0.13	11.77
	Inferior Parietal Lobule 2	0.04	11.70
	Caudate	0.05	11.06
	Angular Gyrus	0.10	10.59
**FreeSurfer**	Left Pars Triangularis Cortical Volume	0.10	12.64
	Right Pars Triangularis Cortical Volume	0.11	11.19
	Right Postcentral Cortical Thickness	-0.11	10.42
	Left Postcentral Cortical Thickness	-0.08	9.34
	Right Banks of Superior Temporal Sulcus Cortical Volume	-0.08	9.06
**sFNC**	Postcentral Gyrus, Postcentral Gyrus	0.08	34.37
	Paracentral Lobule, Superior Parietal Lobule	0.02	18.25
	Inferior Parietal Lobule, Left Inferior Parietal Lobule	0.01	18.02
	Superior Medial Frontal Gyrus, Inferior Parietal Lobule	-0.02	17.55
	Postcentral Gyrus, Precentral Gyrus	-0.06	16.63

**Environmental Factors**	Household Income	-0.10	64.16
	Area Deprivation	0.06	49.04
	School Safety	-0.06	15.91
	Neighborhood Safety	0.004	15.34
	ELS	0.01	8.97

4. We conducted linear mixed effects regression analyses on our 20% test data to examine associations with cognitive measures **(NIH Toolbox Fluid Cognition Composite Score, Crystallized Cognition Composite Score, and Total Cognition Composite Score)**. The cognitive score was the dependent variable, while age, gender, and brain-related canonical variables were fixed effect independent variables. ABCD Family ID nested within ABCD site information was included as a random effect variable.

Supplementary Table 9: Association analyses between cognitive scores and brain-related canonical variables on test data.

	**Canonical Variate**	**Total Composite Score**	**Fluid Composite Score**	**Crystallized Composite Score**
		**(z, p, q)**	**(z, p, q)**	**(z, p, q)**
**Set 1**	**ICA GM**	8.165, p<1e-16, q<1e-16	5.451, p<1e-16, q<1e-16	9.009, p<1e-16, q<1e-16
	**FreeSurfer**	8.081, p<1e-16, q<1e-16	5.214, p<1e-16, q<1e-16	9.189, p<1e-16, q<1e-16
	**sFNC**	8.096, p<1e-16, q<1e-16	5.973, p<1e-16, q<1e-16	7.987, p<1e-16, q<1e-16
**Set 2**	**ICA GM**	-3.366, 0.05, 0.07	-2.202, 0.028, 0.06	-2.810, 0.05, 0.07
	**FreeSurfer**	-2.849, 0.04, 0.07	-1.654, 0.098, 0.12	-2.555,0.021,0.06
	**sFNC**	-0.199, 0.842, 0.84	0.641, 0.52, 0.55	-0.337, 0.736, 0.76
**Set 3**	**ICA GM**	-2.787, 0.05, 0.07	-2.660, 0.08, 0.10	-1.100, 0.271, 0.30
	**FreeSurfer**	-1.827, 0.068, 0.09	-1.516, 0.130, 0.15	-1.280, 0.20, 0.23
	**sFNC**	-3.234, 0.02, 0.06	-4.114, 0.028, 0.06	-1.634,0.102, 0.13
**Set 4**	**ICA GM**	-3.353, 0.031, 0.06	-2.502, 0.02, 0.06	-3.440,0.03, 0.06
	**FreeSurfer**	-2.197, 0.028,0.06	-0.947, 0.344, 0.38	-3.102,0.02, 0.06

	**sFNC**	-3.003, 0.03, 0.06	-2.229, 0.026, 0.06	-2.856, 0.04, 0.06

*q (adjusted p-values -FDR corrected)
